# Demographic and Health Related Data of Users of a Mobile Application to Support Drug Adherence is Associated with Usage Duration and Intensity

**DOI:** 10.1371/journal.pone.0116980

**Published:** 2015-01-28

**Authors:** Stefan Becker, Christopher Brandl, Sven Meister, Eckhard Nagel, Talya Miron-Shatz, Anna Mitchell, Andreas Kribben, Urs-Vito Albrecht, Alexander Mertens

**Affiliations:** 1 Department of Nephrology, University Duisburg-Essen, Essen, Germany; 2 Institute for Drug Safety, University Hospital Essen, Essen, Germany; 3 Institute of Industrial Engineering and Ergonomics of RWTH Aachen University, Aachen, Germany; 4 Fraunhofer Institute for Software and Systems Engineering, Dortmund, Germany; 5 Center for Medicine in the Public Interest, New York City, New York, United States of America; 6 Marketing Department, Business School, Ono Academic College, Kiryat Ono, Israel; 7 Peter L. Reichertz Institute for Medical Informatics (OE8420), University of Braunschweig—Institute of Technology and Hannover Medical School, Hannover, Germany; Gentofte University Hospital, DENMARK

## Abstract

**Purpose:**

A wealth of mobile applications are designed to support users in their drug intake. When developing software for patients, it is important to understand the differences between individuals who have, who will or who might never adopt mobile interventions. This study analyzes demographic and health-related factors associated with real-life “longer usage” and the “usage-intensity per day” of the mobile application “Medication Plan”.

**Methods:**

Between 2010-2012, the mobile application “Medication Plan” could be downloaded free of charge from the Apple-App-Store. It was aimed at supporting the regular and correct intake of medication. Demographic and health-related data were collected via an online questionnaire. This study analyzed captured data.

**Results:**

App-related activities of 1799 users (1708 complete data sets) were recorded. 69% (1183/1708) applied “Medication Plan” for more than a day. 74% were male (872/1183), the median age 45 years. Variance analysis showed a significant effect of the users´ age with respect to duration of usage (p = 0.025). While the mean duration of use was only 23.3 days for users younger than 21 years, for older users, there was a substantial increase over all age cohorts up to users of 60 years and above (103.9 days). Sex and educational status had no effect. “Daily usage intensity” was directly associated with an increasing number of prescribed medications and increased from an average of 1.87 uses per day and 1 drug per day to on average 3.71 uses per day for users stating to be taking more than 7 different drugs a day (p<0.001). Demographic predictors (sex, age and educational attainment) did not affect usage intensity.

**Conclusion:**

Users aged 60+ as well as those with complicated therapeutic drug regimens relied on the service we provided for more than three months on average. Mobile applications may be a promising approach to support the treatment of patients with chronic conditions.

## Introduction

Patients with chronic illnesses such as hypertension and chronic kidney disease are often burdened by high comorbidity and reduced awareness of their medical conditions, which creates a challenging environment in which to promote medication compliance [1]. Complexities of daily life, shifting priorities, and frequent poly-pharmacy likely contribute to patients’ inability to deal adequately with their medical conditions. Frequent encounters with the medical system, which result in dosage adjustments, add to the problems with medication compliance in these patients [2]. For the individual, non-adherence is associated with a number a safety issues such as increased risk of toxicity or more severe relapses [3]. For the health system in Germany alone, direct and indirect costs of non-adherence amount to approximately 7.5 to 10 billion Euros every year [4,5]. Therefore novel strategies are required to address the needs of chronically ill patients. Mobile information technology may offer new system solutions to better meet these requirements. With more than 1 billion users having access to mobile broadband internet and a rapidly growing mobile app market, all stakeholders involved have high hopes that this technology may improve health care [6,7]. Expectations range from overcoming structural barriers to access in low-income countries to more effective, interactive treatment of chronic conditions. Yet previous work suggests that even when sophisticated technology is available, older users (e.g., age 50 and above) find their initial experiences with medication applications frustrating [8]. The iNephro study introduced a “native” smartphone application (“Medication Plan”), which allowed users to maintain and alter personal drug therapy plans and document vital signs on their personal device [9]. In our first paper, we described the implementation of the iNephro Medication Plan in a representative German population subset in the first 15 months after its launch [9]. Our initial findings showed that the regular use of the application decreased considerably within the first 2 months. This paper presents a further analysis to better understand what is generally referred to as “attrition of app usage”. Pre-specified endpoints were used to identify user data with regards to demographic- and health-related factors associated with “duration of usage” and “intensity of usage per day” of the mobile application “Medication Plan”.

## Materials and Methods

### Ethics

The Ethics Committee of the Medical Faculty of Essen University was consulted and a formal written waiver for the need of ethics approval was issued (13-5373-BO).

**Fig 1:**
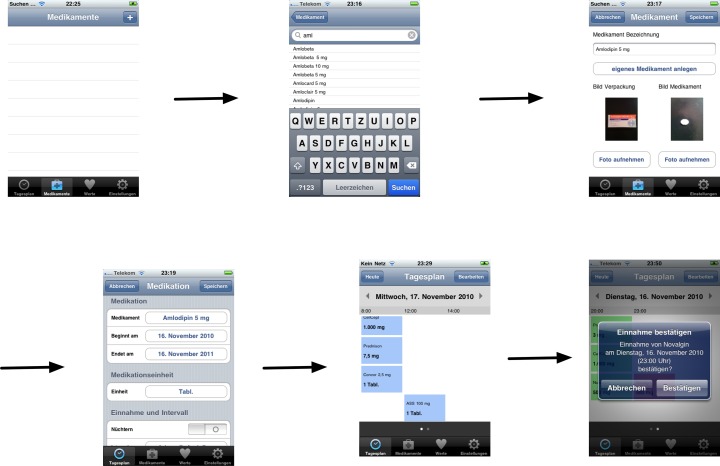
Generating a medication plan on the smartphone [9].

The Department of Nephrology, Essen University Hospital, Essen, Germany, developed and provided the “Medication Plan” application for the iOS platform in 2010 (Becker 2013). This native smartphone application allowed users to maintain and alter a drug therapy plan on their personal device [9,10] (Fig. 1). Between December 2010 and January 2012 it was available free of charge for download in the German-language App Store by Apple. Due to a lack of funding required updates could not be realized and the app had to be taken out of the App Store in December 2013.

Users were able to specify intake requirements according to the medication regimen issued by the prescribing physician and the patients’ own personal needs (see tutorial at http://www.youtube.com/watch?v=nui78JqwMHE). A reminder function and local push-notification alerts reminded users to take their medications at the pre-specified time. No permanent internet connection was necessary and all data was stored locally on the device itself, reducing the possibility of erroneous transmission of personal health information. Users could enter vital sign data, and trends were presented graphically. Prior to using the application, users had to give their consent to a disclaimer as well as an agreement for a subsequent anonymous analysis of user data and this was done via activation of a hyperlink (implementation by QUEST objects GmbH, Tübingen). Additionally, users were invited to voluntarily and anonymously complete an online questionnaire, which had to be actively accessed via an additional hyperlink. The unique identifier numbers (UDID) of the respective iPhones were irreversibly encrypted by a MD5 message-digest algorithm (MD5-Hash). The activity of the encrypted UDID was then tracked (“creation”, “modification”, or “deletion” of drug information, as well as the “confirmation” of drug adherence within the application “Medication Plan”). Activity of the respective, encrypted UDID addresses and if available, associated demographic information, were analyzed using self-implemented software of Fraunhofer ISST, Dortmund, Germany. Further statistical analysis was performed using SPSS 21.0 (IBM, U.S.A.): A multi-factorial analysis of variance (ANOVA) for the response variables with a significance level of 0.05 was conducted. The distributions of the residuals were normal and the homogeneity of variances for the data analyzed was confirmed. Following the APA guidelines the exact error probability p was specified for each analysis [11]. Dependent variables were “duration of longer usage” (defined as use of >1 day; if no activity was recorded for > 10 days, this was rated as end of use) and “mean intensity of usage per day” during time of active usage.

## Results

**Fig 2:**
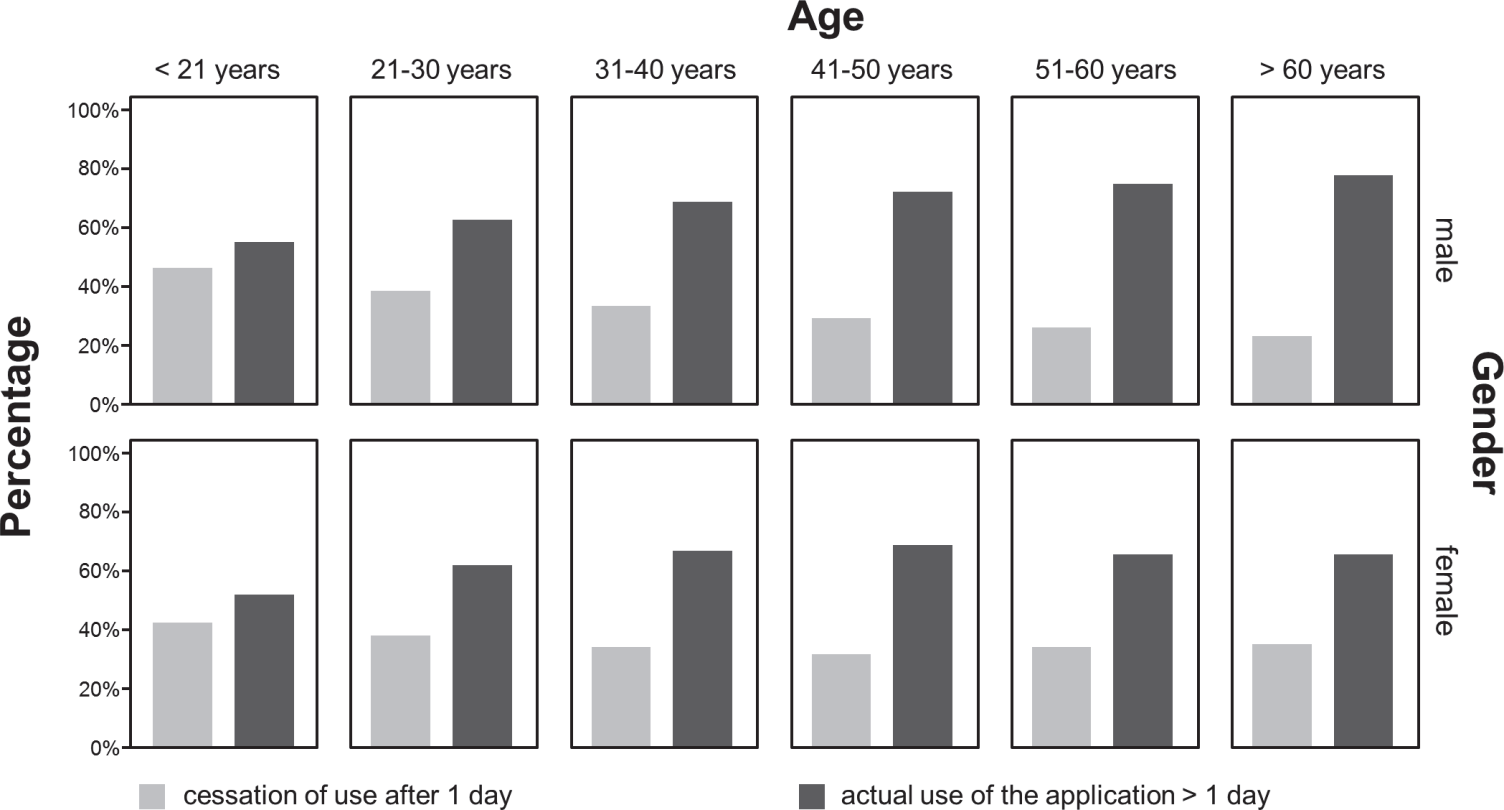
Distribution of user behavior: actual use of the application > 1 day and cessation of use after 1 day, by age and sex.

**Fig 3:**
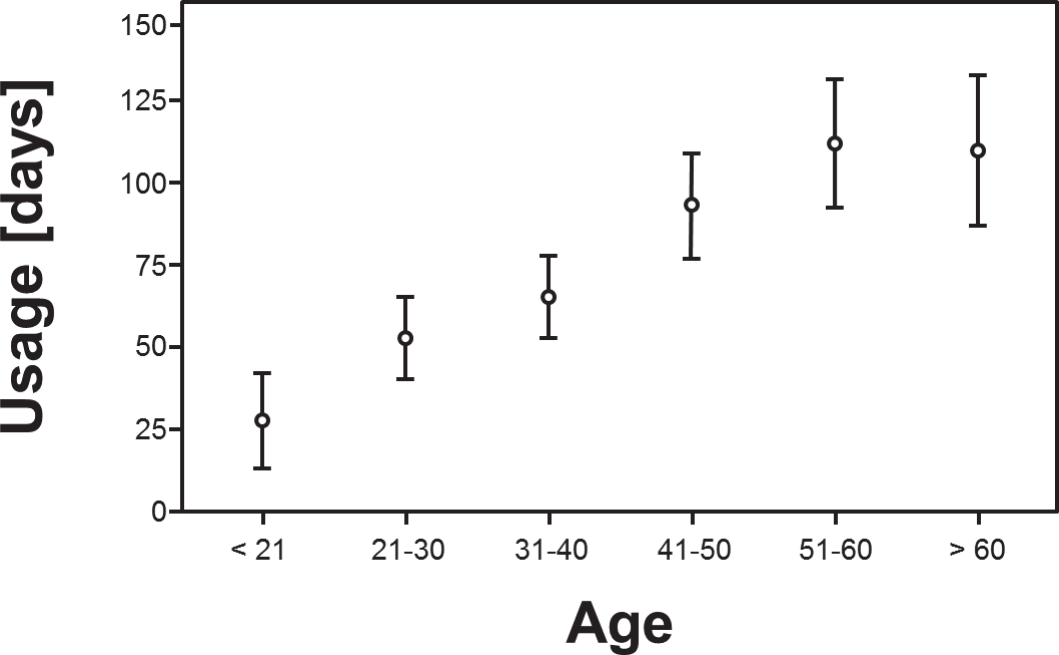
There was a significant increase of mean usage duration between the age cohorts < 21 and >60 years (F = 2.581; df = 5; p = 0.025).

**Fig 4:**
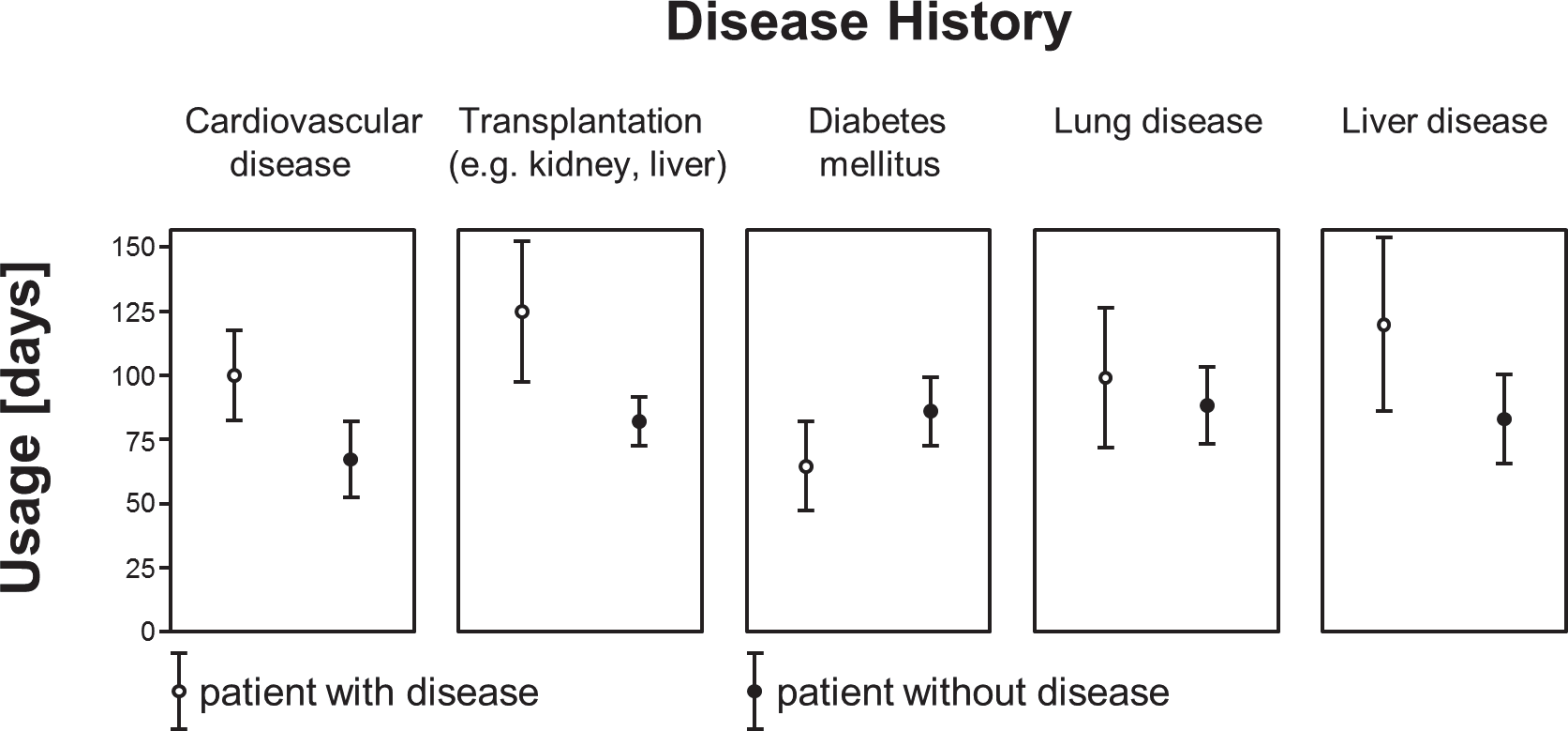
Specific diseases are associated with a longer usage of the application.

**Fig 5:**
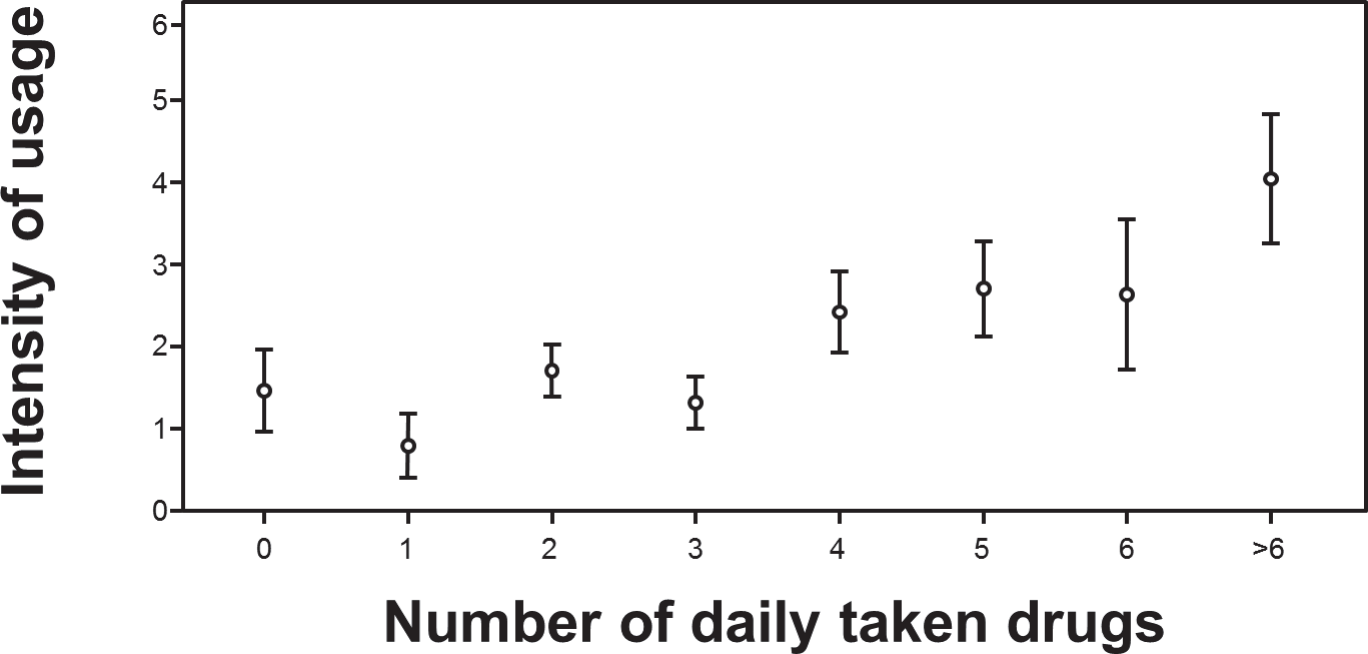
The number of regularly taken drugs had significant impact on usage intensity (F = 4.017; df = 7; p < 0.001).

Overall, activity of 1799 (1708 complete data sets) users was recorded between December 2010 and April 2012 (Table 1). More than two thirds of users (1183/1708) used “Medication Plan” for more than a day. Overall, men used the application significantly longer than women (χ² (1, N = 1761) = 6.715, p < 0.010). Looking at the different age cohorts, there was a significant correlation between age and “longer usage” (> 1 day) (χ² (5, N = 1799) = 15.255, p < 0.001). With rising age, the reliance on the application for more than 1 day rose from 50% of those below 21 years of age to over 70% for those aged 40 years or older, yet the effect was stronger in men for all age cohorts (Fig. 2). The number of diseases for each user significantly affected duration of usage (χ² (5, N = 1799) = 12.144, p = 0.030). While the proportion of individuals who stopped using the app after one day was 42% for those that did not have to take any drugs, it was between 25 and 30%, who were on regular medication. Similarly the number of drugs significantly affected the duration of usage (χ² (7, N = 1799) = 30.612, p < 0.001). This effect is not surprising, as for users without any medical condition or the need to take drugs on a regular schedule, only a fraction of the available functions was still useful (e.g. keeping track of weight and vital parameters). Variance analysis presented the following effects with respect to duration of usage: With a mean duration of usage of 23.3 days (SD 36.9) by users < 21 years, there was a substantial increase over all age cohorts up to users of 60 years and above using the application for 103.9 days on average (SD 20.7) (F = 2.581; df = 5; p = 0.025). For users aged 50 and older, the usage duration seemed to remain static. Post hoc pairwise analysis with Bonferroni correction showed significant differences between all groups with a minimum age difference of 20 years. I.e. users aged 50 used the app substantially more than those aged 30. Mean duration of usage, for users who did not abandon the application within the first day, was 85.4 days (SD 138.6) (Fig. 3). Sex (F = 1.084; df = 1; p = 0.298) and educational attainment (F = 0.656; df = 2; p = 0.519) had no effect for those that did not cease to use the application after one day. The number of medical conditions (F = 0.403; df = 5; p = 0.847) as well as the number of drugs taken per day on a regular schedule (F = 0.967; df = 7; p = 0.259) did not affect the duration of usage significantly. Interestingly, variance-analysis of the individual medical conditions showed a significant effect on duration of usage if the user suffered from cardiovascular disease (F = 14.098; df = 1; p < 0.001) or had received a transplant (F = 12.503; df = 1; p < 0.001) (Fig. 4). In either case, people with these diseases used the system on average about 50% longer compared to people not suffering from the same condition. Diabetes (F = 2.699; df = 1; p = 0.101), lung disease (F = 0.411; df = 1; p = 0.522) and liver disease had no significant impact on duration of usage (F = 2.221; df = 1; p = 0.136). With regard to usage intensity, the number of diseases tended to affect usage intensity (F = 1.974; df = 5; p = 0.080). The number of regularly taken drugs had significant impact on usage intensity (F = 4.017; df = 7; p < 0.001) and increased with the number of drugs taken per day (Fig. 5). Demographic predictors such as sex (F = 0.874; df = 1; p = 0.350), age (F = 0.646; df = 5; p = 0.665) and educational attainment (F = 0.905; df = 2; p = 0.405) showed no significant effect.

**Table 1:** Characteristics of the analyzed cohorts.

Variable		overall (n = 1708)		< 1 day users (n = 525)		> 1 day users (n = 1183)	
Sex	Male	1225	72%	353	67%	872	74%
	Female	483	28%	172	33%	311	26%
Age (years)	<21	45	3%	21	4%	24	2%
	21–30	254	15%	96	18%	158	13%
	31–40	354	21%	112	21%	242	20%
	41–50	447	26%	122	23%	325	27%
	51–60	343	20%	98	19%	245	21%
	>60	265	16%	76	14%	189	16%
Highest educational qualification	Finished secondary school	904	53%	274	52%	630	53%
	Finished school with qualifications for university studies	328	19%	113	22%	215	18%
	Holding a university degree	476	28%	138	26%	338	29%
Disease	Cardiovascular diseases	894	52%	243	46%	651	55%
	History of transplantation (e.g. kidney or liver)	243	14%	74	14%	169	14%
	Diabetes mellitus	125	7%	46	9%	79	7%
	Lung disease	86	5%	22	4%	64	5%
	Liver disease	90	5%	32	6%	58	5%
Number of chronic conditions	0	495	29%	174	33%	321	27%
	1	806	47%	230	44%	576	49%
	2	280	16%	83	16%	197	17%
	3	95	6%	27	5%	68	6%
	4	24	1%	7	1%	17	1%
	5 and more	8	0%	4	1%	4	0%
Number of daily taken drugs	0	212	12%	90	17%	122	10%
	1	397	23%	121	23%	276	23%
	2	330	19%	85	16%	245	21%
	3	257	15%	74	14%	183	15%
	4	165	10%	39	7%	126	11%
	5	149	9%	54	10%	95	8%
	6	91	5%	31	6%	60	5%
	7 and more	107	6%	31	6%	76	6%

## Discussion

Our study is the first to describe the acceptance of a medication adherence tool in a representative German population subset. More than 2/3 of users continued using “Medication Plan” after the first day. Over the entire study population, use of the application for more than 1 day grew with increasing age, yet the effect was greater in men over all age cohorts. Once a user decided to log into the application, further factors (disease and associated medication) had an effect on the usage-duration and -intensity. Such findings may provide useful insights for behavioural-patterns of designated patient groups and assist in the design of effective interventions.

### Brief review of findings and comparison to prior studies

Thus far, most users of publicly available health related mobile communication services seem to have been “early adopters”: middle-aged, male, well-educated and comparatively healthy [9, 12–14]. Furthermore, high attrition rates for internet interventions are reported, which may reflect deficits in usability or an early interest in the novelty of the application, with a declining eagerness as the newness of the intervention wears off [9, 15, 16]. The question that arises is whether mobile technology to support drug adherence is indeed an appropriate tool to support elderly patients with polypharmacy. So far, this issue has mostly been assessed indirectly via questionnaires [17–20]. Direct assessment of user activity can offer objective and more accurate data on the usefulness of a mobile application. Our findings offer new insights into what has been termed the “digital divide” [13, 14], a term coined by developers, which implies, that compared with the younger generation, older individuals are less likely to make extensive use of digital technology [8, 21]. Whereas our data show a strong effect of the variables “age” and “disease” on longer usage, “polypharmacy” was identified as the primary influence for the intensity of usage. No effects were identified for educational status. Our findings contradict any generalized assumption implying "digital disengagement of the elderly". We have shown that for smartphone users, “increasing age” or “polypharmacy” are good predictors for acceptance of mobile technology when it comes to supporting adherence. The findings lead to the question whether drug adherence can eventually be improved in users of such technology. The Technology Acceptance Model suggests that acceptance depends on a user’s perception of the usefulness and ease of use of a system [22]. Similarly, the diffusion of innovation model emphasizes that a new technology needs to offer a “relative advantage” over the status quo [23]. Together with previous findings suggesting that increasing age [24–26] and polypharmacy [27] negatively affect adherence, one may assume that the use of applications like "Medication Plan" will eventually improve drug adherence. In our investigation the lack of perceived usefulness or relative advantage likely was the reason for relative early cessation of app usage by patients stating to be suffering from diabetes is a point in proof [28]. “Medicaton Plan” did not offer many of these users the required added value such as a combined documentation of blood sugar, bread units consumed or administered doses of insulin. This finding demonstrates how important it is to bear in mind specific needs of users to achieve acceptance [29]. Furthermore, the success of "Medication Plan" with its very practical aspects of drug regimen management supports the concept of 'small data' [30], suggesting that people mainly need technology to help them make sense of their health condition, and to offer actionable steps. On another note, even though the three main drivers of adherence to chronic disease medication were identified as perceived concerns about medications, perceived need for medications, and perceived affordability of medications [31], it appears that practical support and reminders, while not directly targeting those issues, can still go a long way toward self-reported drug adherence. Lastly it seems, that the focus of iNephro on practicality and its main purpose to help users overcome the current barriers to health literacy, were recognized and are mirrored by our results [32].

### Strengths and limitations

Despite evidence from pilot studies, most mHealth interventions can be seen as the equivalent of black boxes [19]: The problem of these studies is that a particular style of a black box application is compared to a situation without any black box application. This study presents a novel approach to directly assess usage and acceptance of mobile technology in the context of drug adherence. Our elderly iPhone users can be seen as technologically savvy in the first place and findings may not apply across the entire population [33]. Age-related changes in hearing, vision, cognition, and mobility require special consideration and application designers need to take this into account. Elderly patients tend to be more reserved towards the use of technology since they usually feel barriers to start using it [34]. Unless they see a clear benefit for themselves, older adults are less likely to adopt new technology [28].

### Conclusion

The famous statement by Dr. Everett Koop, former U.S. Surgeon General, “Drugs don’t work in patients who don’t take them” may also be true for mobile medication management applications [8]. We were able to show that elderly, technologically savvy users requiring polypharmacy, relied on a mobile application to support drug adherence and that the degree of engagement depended on disease/therapy-related as well as demographic factors. With this knowledge, similar tools could be valuable in the drug management of these patients. However, particularly in elderly patients, drug adherence is a complex problem, requiring not only a trust based doctor-patient relationship but rather multidimensional approaches. These range from simplified therapy-regimes and sustained understanding of the disease on the patients’ side to technological support including companion pillbox and communication devices. Applications will have to be tailored closely to the specific demands of sick individuals to be accepted as part of their often complicated day-to day routines [35, 36]. Hence, interdisciplinary approaches and a profound understanding of the context and patients’ needs are vital for successful realization of technological solutions and investments in the field [7, 37].
